# Quorum sensing inhibition strategies for the control of *Pseudomonas aeruginosa* infections

**DOI:** 10.1007/s11274-026-05014-9

**Published:** 2026-06-10

**Authors:** Juan Sánchez-Mateos, José David Flores-Félix

**Affiliations:** 1 Departamento de Bioquímica y Biología Molecular, Universidad de Salamanca, Instituto de Investigación Biomédica de Salamanca (IBSAL), Salamanca, 37007 Spain; 2 Department of Microbiology and Genetics, Edificio Departamental de Biología, University of Salamanca, Salamanca, 37007 Spain; 3 Institute for Agribiotechnology Research (CIALE), Salamanca, 37185 Spain; 4 Research Unit of Excellence “Agricultural Production and Environment” (AGRIENVIRONMENT), University of Salamanca, Salamanca, 37185 Spain

**Keywords:** *Pseudomonas aeruginosa*, Quorum quenching, Antimicrobial strategies, Antivirulence therapy, Biofilm inhibition

## Abstract

*Pseudomonas aeruginosa* is an opportunistic pathogen responsible for severe and persistent infections, especially in immunocompromised patients. Its high adaptability and antibiotic resistance are largely mediated by quorum sensing (QS), a cell-to-cell communication system that coordinates the expression of virulence factors and biofilm formation. Targeting QS—rather than bacterial viability—has emerged as a promising antivirulence strategy known as quorum quenching (QQ). This review analyses current QQ approaches against *P. aeruginosa*, emphasizing their mechanisms, efficacy, and translational potential. Five main strategies are discussed: (i) inhibition of autoinducer synthesis through enzyme blockers targeting LasI, RhlI, and Pqs biosynthetic enzymes; (ii) enzymatic degradation or inactivation of QS signals by lactonases, acylases, and oxidoreductases; (iii) interference with signal release, particularly via disruption of outer membrane vesicle formation; (iv) inhibition of QS receptors (LasR, RhlR, and PqsR) using natural and synthetic antagonists; and (v) suppression of downstream signalling cascades. Although significant advances have been achieved, the clinical translation of QS inhibitors remains limited by issues of molecular stability, strain variability, and bioavailability. Nevertheless, structure-guided inhibitor design, synergistic combinations with antibiotics, and biofilm-targeted delivery systems are expanding the therapeutic landscape. Overall, QQ represents a sustainable strategy to attenuate bacterial virulence while minimizing selective pressure for resistance, offering a paradigm shift in the treatment of *P. aeruginosa* infections and other multidrug-resistant pathogens.

## Introduction

Since the discovery of antibiotics in the mid-20th century, these drugs have constituted the cornerstone of therapy against bacterial infections. However, their continuous—and often inappropriate—use has promoted the emergence and dissemination of antimicrobial resistance, representing one of the most critical global health challenges today. Recent global estimates indicate that antimicrobial resistance was associated with approximately 4.71 million deaths and directly responsible for 1.14 million deaths worldwide in 2021, with projections suggesting that this burden could rise to nearly 1.91 million attributable deaths annually and more than 8 million associated deaths by 2050 if effective interventions are not implemented (Naghavi et al. 2024).

In this context, *Pseudomonas aeruginosa*, a Gram-negative opportunistic bacterium, has been classified by the WHO as a critical-priority pathogen for the development of new antibiotics (World Health Organization, 2024). This classification arises from its remarkable metabolic versatility and ability to colonize diverse environments, including hospital settings (Ambreetha et al. 2024), where it acts as an etiological agent of severe nosocomial infections—particularly in immunocompromised patients or those with implanted medical devices. Clinically, it can cause acute and chronic infections in the respiratory and urinary tracts, bloodstream, surgical wounds, and burn sites (Reynolds and Kollef 2021).

During infection, the bacterium secretes a wide variety of virulence factors that enable adhesion, invasion, and colonization of host tissues, while also evading immune defenses (Table 1). These factors are generally grouped into functional categories: **(i)** degradative enzymes, which promote tissue destruction and dissemination; **(ii)** toxins and secondary metabolites, which alter cellular physiology and modulate immune responses; and **(iii)** nutrient acquisition systems, such as siderophores, which enable survival under iron-limited conditions (Chadha et al. 2022; Liao et al. 2022; Qin et al. 2022). Multiple studies have demonstrated that the expression of these factors is closely regulated by bacterial communication systems, making these mechanisms highly attractive therapeutic targets (Filloux and Ramos 2022; Miranda et al. 2022).

**Table 1:** Virulence factors of *P. aeruginosa*

Functional category	Virulence factor	Main function	Role in pathogenesis and disease severity	Reference
Host colonization and bacterial motility	Chemotaxis receptors	Enable directed bacterial motility toward chemical gradients.	Facilitate movement toward damaged epithelial areas, promoting colonization	Schwarzer et al. 2016
	Histamine-mediated chemotaxis receptors	High-affinity chemoreceptors for histamine	Stimulate histamine metabolism and bacterial motility, enhancing QS activation and QS-dependent virulence factors.	Krell et al. 2021
	Phosphate gradient-mediated chemotaxis receptors	Detect and respond to low-phosphate conditions	Contribute to phosphate uptake and activate QS pathways that increase the expression of virulence genes	Rico-Jiménez et al. 2016
Extracellular invasive enzymes	Alkaline protease	Degrades transferrin, complement proteins, cytokines, and structural components of the extracellular matrix	Increases iron availability, induces tissue damage and necrosis, and contributes to immune evasion	Heck et al. 1986; Matheson et al. 2006; Laarman et al. 2012
	LasA Protease	Enhances the elastolytic activity of LasB and cleaves glycine-rich proteins	Disrupts epithelial barriers, promoting bacterial invasion, cell damage, and immune evasion	Kessler et al. 1997
	LasB Elastase	Catalyzes proteolytic cleavage of extracellular matrix proteins and immune components.	Facilitates iron acquisition, causes severe tissue damage, and promotes invasion and infection	Everett and Davies 2021
	Phospholipases	Hydrolyze phospholipids from cellular membranes	Induce hemolysis and apoptosis, facilitating pathogen dissemination	Vasil et al. 1991; Kirschnek and Gulbins 2006
Toxic secondary metabolites	Hydrogen cyanide	Inhibits cytochrome c oxidase, blocking cellular respiration	Contributes to hypoxia and cell death in pulmonary infections, leading to reduced lung function	Lenney and Gilchrist 2011
	Lectins A and B	Bind to glyco-conjugates on epithelial cells	Paralyze respiratory cilia, stabilize biofilm formation, and promote colonization, impairing respiratory processes	Diggle et al. 2006; Passos da Silva et al. 2019
	Pyocyanin	Inhibits cellular respiration by inducing oxidative stress and cytotoxicity through generation of reactive oxygen species (ROS) such as hydrogen peroxide (H₂O₂) and superoxide anion (O₂⁻)	Promotes infection establishment and progression, facilitates bacterial colonization, paralyzes respiratory cilia, causes tissue damage, and supports immune evasion	Hall et al. 2016
	Ramnolípidos	Biosurfactants that cause hemolysis and death of macrophages and neutrophils, inhibit ciliary function in the respiratory epithelium, and impair mucociliary clearance	Accelerate tissue invasion and damage, infection establishment, and immune evasion, while reducing antibiotic penetration.	Zulianello et al. 2006; Abdel-Mawgoud et al. 2010
Siderophores	Pyochelin	Iron chelator with moderate affinity for iron in the host microenvironment and from lysed erythrocytes	Facilitates bacterial growth, invasion, and biofilm development	Cornelis 2010
	Pyoverdine	Iron chelator with high affinity for iron from the host microenvironment and lysed erythrocytes	Facilitates bacterial growth, invasion, and biofilm development	Cornelis 2010)
Toxins	Exotoxin A	Inhibits host protein synthesis by ADP-ribosylating the elongation factor eEF-2	Causes local immunosuppression, significant tissue damage, and cell death	Michalska and Wolf 2015
Biofilm formation	Alginate	Essential biofilm component that protects bacteria from host immune responses	Promotes immune evasion and protects the pathogen from ROS and antibiotics	Brindhadevi et al. 2020; Thi et al. 2020
	Rhamnolipids	Biosurfactants that maintain open channels within biofilms and modulate intercellular communication	Support biofilm architecture and reduce antibiotic penetration	Brindhadevi et al. 2020; Thi et al. 2020

Importantly, several virulence factors also play direct roles in biofilm formation and maintenance, as summarized in Table 1, highlighting the functional overlap between pathogenicity and persistence mechanisms. In this context, biofilm formation constitutes one of the main strategies underlying resistance and long-term persistence in *P. aeruginosa*. Biofilms are three-dimensional communities of bacterial cells embedded in an extracellular matrix composed mainly of exopolysaccharides, proteins, and extracellular DNA. This organization not only acts as a physical barrier against antibiotics and host immune defenses but also represents an adaptive strategy that facilitates survival under adverse environmental conditions, colonization of both biotic and abiotic surfaces, and the establishment of chronic infections that are difficult to eradicate (Flemming et al. 2016; Brindhadevi et al. 2020; Thi et al. 2020).

**Fig. 1:**
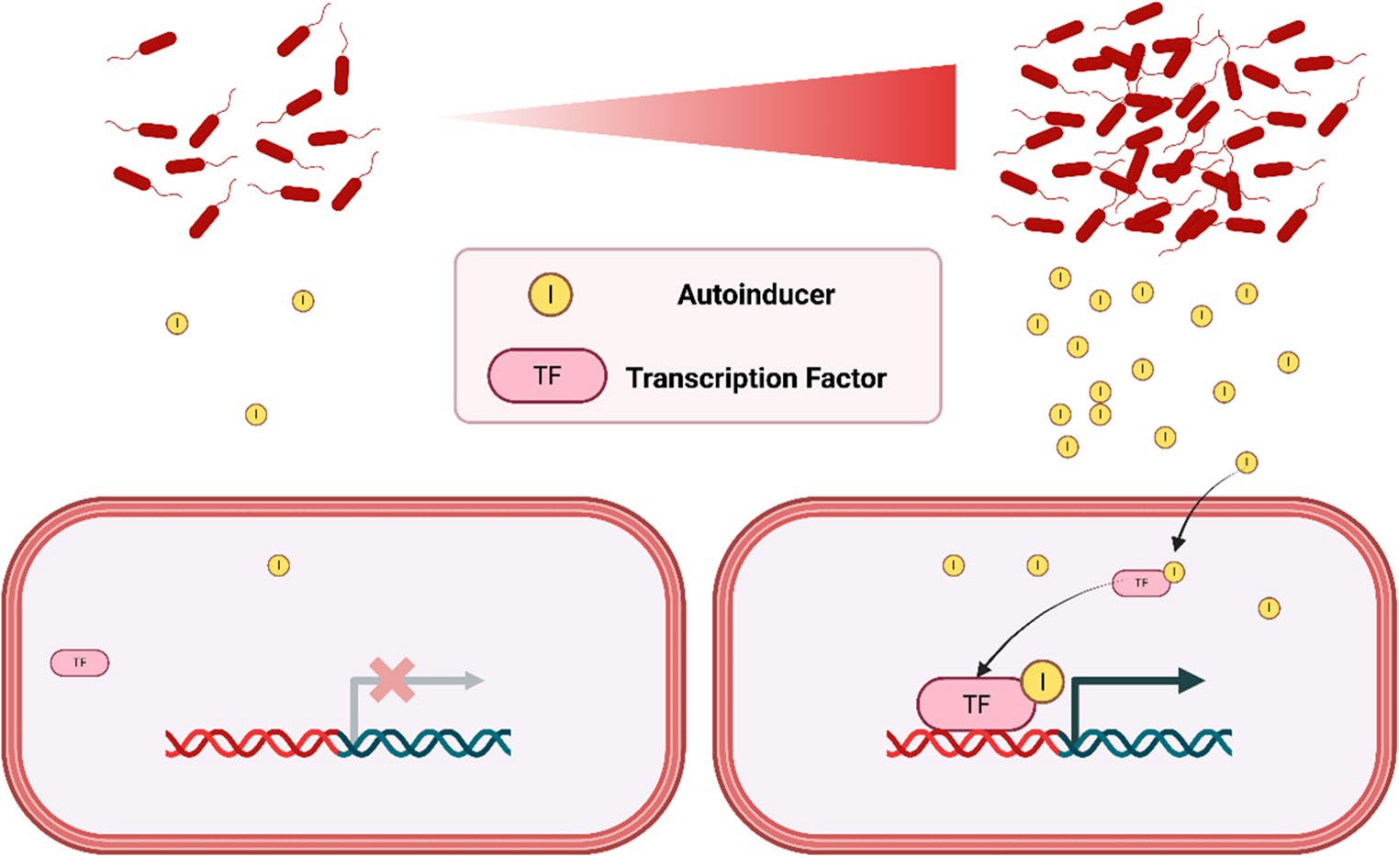
General mechanism of a QS system. Original figure created with BioRender.com

Together, virulence factor production and biofilm formation drive the transition from acute to chronic infection, representing tightly interconnected processes that enhance bacterial persistence. Most virulence factors of *P. aeruginosa*, as well as biofilm formation, are under the control of an intercellular communication system known as quorum sensing (QS). This system relies on the synthesis and release of signaling molecules known as autoinducers, which accumulate in the environment until a threshold concentration is reached. Once detected by specific receptors, they trigger signaling cascades that collectively regulate gene expression (Filloux and Ramos 2022; Vadakkan et al. 2024a), as illustrated in Fig. 1. This coordination enables the bacterium to synchronize key processes related to virulence, environmental adaptation, and persistence, thereby contributing to its success as an opportunistic pathogen (Moradali et al. 2017).

From a therapeutic standpoint, QS represents a strategic target for the control of *P. aeruginosa* infections. Inhibition of this system, known as quorum quenching (QQ), does not directly affect bacterial viability but rather interferes with its ability to coordinate the expression of virulence factors and to form biofilms. This approach, which targets nonessential processes for bacterial survival, is particularly attractive because it reduces the selective pressure that typically promotes the emergence of resistance, a recurrent issue with traditional antibiotics. Moreover, several studies have demonstrated that QS inhibition can enhance the efficacy of conventional antibiotics by increasing biofilm susceptibility, paving the way for potentially more effective combination therapies (Duplantier et al. 2021; Li et al. 2022; Rather et al. 2022; Rodriguez-Urretavizcaya et al. 2024). In this scenario, QQ emerges as a promising, currently developing strategy with high potential to complement existing antibiotics and improve clinical outcomes in infections associated with *P. aeruginosa*.

The objective of this review is to analyze the different QQ strategies in *P. aeruginosa* and to evaluate their potential as therapeutic alternatives against infections caused by this pathogen. The review aims to integrate up-to-date information on the mechanisms of action of various inhibitors, their efficacy in experimental models, and the potential limitations and challenges for their clinical application.

## Materials and methods

A structured literature review focused on QS and QQ strategies in *P. aeruginosa* was conducted, incorporating elements of the PRISMA statement to enhance transparency and reproducibility.

## Search strategy

A comprehensive literature search was performed using PubMed and Web of Science databases. The search included articles published up to 2025, with particular focus on studies from 1990 onwards to capture both foundational and recent advances in QS research.

The search strategy combined the following keywords and Boolean operators: “*Pseudomonas aeruginosa*”; “quorum sensing”; “quorum quenching” OR “quorum sensing inhibition”; “biofilm”; “virulence factors”; “anti-virulence”; “signal molecules” OR “autoinducers”; “LasR”, “RhlR”, “PqsR”.

Additional keywords such as “monoclonal antibody” and “outer membrane vesicles” were included to capture emerging QQ strategies. Search terms were adapted to each database to maximize retrieval. In addition, the reference lists of relevant articles were manually screened to identify further studies not captured in the initial search.

## Eligibility criteria

Studies were included if they: (i) were peer-reviewed articles (original research or reviews); (ii) addressed QS systems, QQ mechanisms, virulence regulation, or biofilm formation in *P. aeruginosa*; (iii) reported experimental (in vitro/in vivo), computational, or mechanistic data;

(iv) were published in English. Studies were excluded if they: (i) were not peer-reviewed (e.g., editorials, conference abstracts); (ii) were duplicates; (iii) did not provide relevant information on QS/QQ in *P. aeruginosa*; (iv) focused exclusively on other microorganisms without transferable insights.

## Study selection process

Articles retrieved from database searches were screened based on title and abstract. Relevant studies were subsequently assessed through full-text review. Selection was based on relevance to QS regulation and QQ strategies in *P. aeruginosa*. Although this study is not a formal systematic review, efforts were made to apply structured and reproducible selection criteria in line with PRISMA Statement recommendations.

### Data extraction and categorization

Relevant information from the selected studies (approximately 600 articles) was extracted and organized into predefined thematic categories corresponding to the main QQ strategies analyzed in this review: (i) inhibition of autoinducer synthesis; (ii) enzymatic degradation or inactivation of QS signals; (iii) interference with signal release; (iv) receptor inhibition and (v) disruption of downstream signaling pathways. Particular attention was given to studies addressing translational aspects, including in vivo efficacy, combinatorial approaches with antibiotics, and potential clinical applicability.

## Data synthesis and management

A qualitative synthesis was performed to integrate findings across studies. Emphasis was placed on identifying conserved mechanisms of QS regulation, experimentally validated QQ strategies, emerging therapeutic approaches, and existing gaps or inconsistencies in literature. All references were managed using Mendeley, and relevant data were compiled into tables and figures to support transparency and reproducibility of the review process.

## Quorum sensing in ***Pseudomonas aeruginosa***

The virulence and environmental adaptation of *P. aeruginosa* are governed by a sophisticated, non-linear regulatory network comprising four interconnected QS systems: Las, Rhl, Pqs, and the stress-integrated IQS (Integrated Quorum Sensing Signal). Rather than operating as isolated pathways, these circuits form a redundant and flexible framework where hierarchy and cross-communication ensure survival and pathogenicity.

**Fig. 2:**
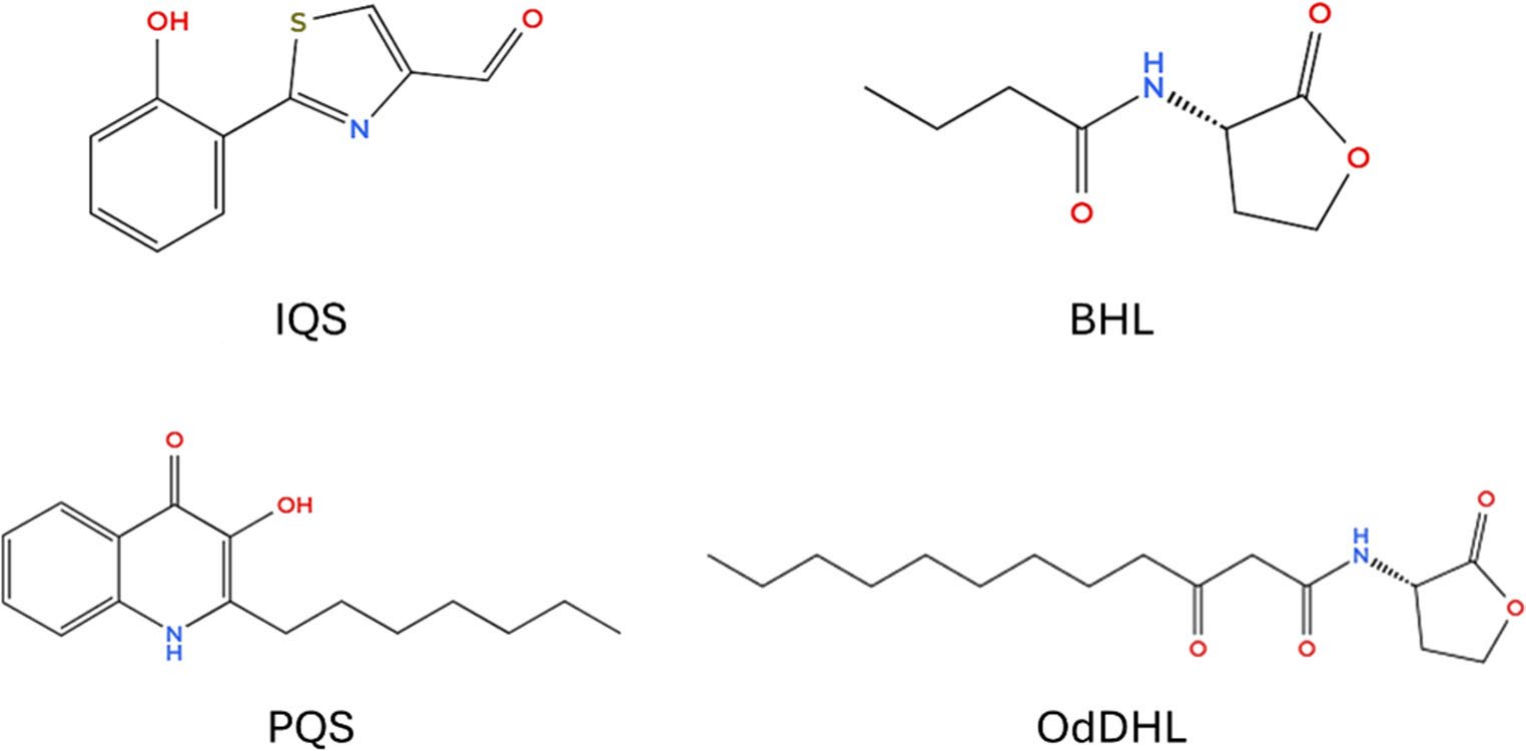
Chemical structures of the QS signaling molecules of *P. aeruginosa*. From upper left: 2-(2-hydroxyphenyl)-thiazole-4-carbaldehyde (IQS); N-butanoyl-L-homoserine lactone (BHL); 4-hydroxy-2-heptyl-4-quinolone (PQS); and N-(3-oxododecanoyl)-L-homoserine lactone (OdDHL)

At the top of the signalling cascade sits the Las system, which acts as a primary coordinator. The synthase LasI produces the signal (*N*-(3-oxododecanoyl)-L-homoserine lactone, OdDHL; see Fig. 2); once it binds to the LasR receptor, the complex triggers the expression of critical virulence factors such as elastase (*lasB*), exotoxin A (*toxA*), and alkaline protease (*aprA*) (Gilbert et al. 2009; Chadha et al. 2022). Moreover, it activates the Rhl and Pqs systems, underscoring the central role of Las in coordinating global virulence (Lee and Zhang 2015; Vadakkan et al. 2024a). Crucially, LasR initiated signalling provides the first level of integration by activating the downstream Rhl and Pqs systems.

The Rhl system—mediated by RhlI-synthesized (activated by the signal *N*-butanoyl-L-homoserine lactone (BHL) and its receptor RhlR—governs factors essential for colonization and biofilm stability, including rhamnolipids (*rhlAB*) (Reis et al. 2011), hydrogen cyanide (*hcnABC*) (Schuster and Greenberg 2007)., and lectins (*lecA* and *lecB*) (Winzer et al. 2000). While LasR initiates this system, RhlR also participates in regulating *lasB* directly or indirectly depending on environmental conditions, demonstrating the network’s early-stage feedback loops (Brint and Ohman 1995; Schuster and Greenberg 2007; Lee and Zhang 2015).

Interwoven with the AHL systems is the Pqs system, controlled by the LysR-type regulator PqsR (or MvfR) (Cao et al. 2001). The *pqsABCDE* operon produces 2-heptyl-4-quinolone (HHQ) (De et al., 2003; Schertzer et al. 2010; Dulcey et al. 2013), which is subsequently converted into the *Pseudomonas* Quinolone Signal (PQS; 4-hydroxy-2-heptyl-4-quinolone**)** by PqsH. Both HHQ and PQS bind to PqsR to establish a positive feedback loop that intensifies the production of pyocyanin (*phzA1-G1/A2-G2*), siderophores like pyoverdine (*pvdA*) and pyochelin (*pchE*), and oxidative stress resistance enzymes (Xiao et al. 2006).

The more recently identified IQS system links this network to nutrient stress, specifically phosphate scarcity. While its receptor remains unknown and its exact biosynthesis, partially attributed to the *ambBCDE* operon, is still debated, IQS is known to modulate both Las and Pqs activities during periods of environmental limitation (Lee et al. 2013; Raya et al. 2025).

**Fig. 3:**
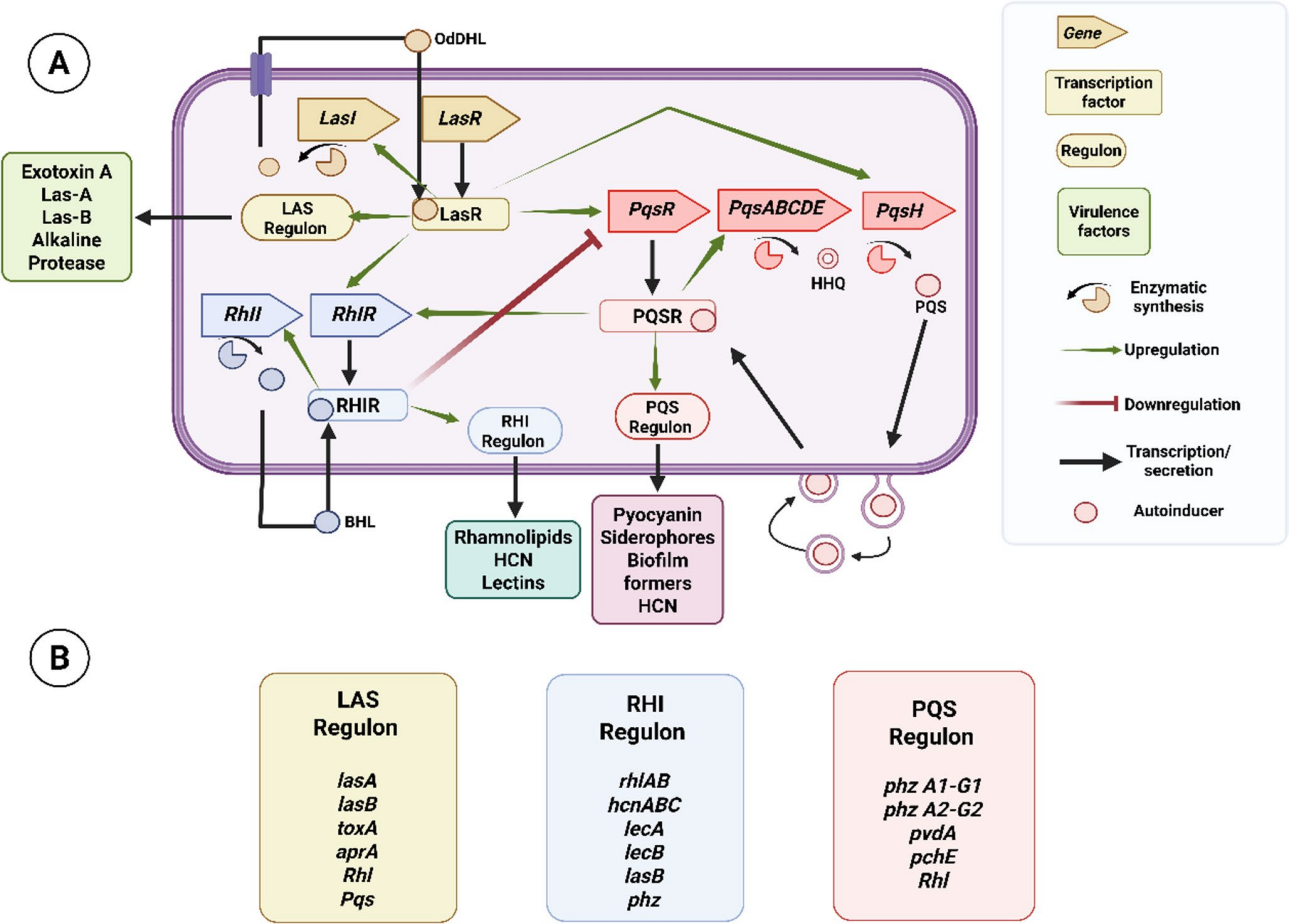
QS signaling systems in *P. aeruginosa*. Original figure created with BioRender.com

The hallmark of *P. aeruginosa* QS is its regulatory plasticity (Fig. 3). A critical feedback interaction exists between Pqs and Rhl: PQS enhances RhlR activity for secondary metabolite production, while RhlR in turn represses PQS overproduction to prevent excessive signalling. This interconnectedness provides a compensatory mechanism where the loss of a “master” regulator like *lasR* does not necessarily abolish virulence, as the Rhl and Pqs circuits can partially maintain the pathogenic profile. This robust architecture presents a significant challenge for clinical intervention, as targeting a single system may be insufficient to overcome the network’s inherent redundancy.

### Quorum quenching strategies

In general terms, QQ can be grouped into five major categories: (i) blockade of autoinducer synthesis, (ii) degradation, inactivation, or sequestration of produced signals, (iii) interference with signal release into the extracellular environment, (iv) specific inhibition of signal receptors, and (v) suppression of downstream signaling cascades following ligand–receptor binding (Zhou et al. 2020; Rather et al. 2022). This classification reflects the diversity of approaches proposed and provides a useful framework to analyze both their therapeutic potential and their inherent limitations. Subsequently, these strategies will be examined in greater detail within the context of the best-characterized QS systems in *P. aeruginosa* (Las, Rhl, and Pqs), allowing assessment of their advantages and challenges in specific infection scenarios.

## Inhibition of autoinducer synthesis

Blocking autoinducer synthesis represents one of the most direct strategies to disrupt bacterial communication, as it targets the initial step of signal transduction. The key enzymes involved include LasI (OdDHL), RhlI (BHL), the ***pqsABCDE*** operon (formation of 2-heptyl-4-quinolone, HHQ), and PqsH (conversion of HHQ into PQS).

## Inhibition of AHL synthesis (LasI and RhlI – AHL synthases)

Acyl-homoserine lactone (AHL) synthases (LasI and RhlI) belong to the LuxI family of enzymes that catalyze the acylation of *S*-adenosylmethionine (SAM) with an acyl-ACP (or acyl-CoA) to form the corresponding *N*-acyl-L-homoserine lactone (Parsek et al. 1999).

A classical approach involves the use of SAM analogs and catalytic intermediates designed to compete with the natural substrate at the active site of AHL synthases Compounds such as S-adenosylhomocysteine (SAH), sinefungin, (Chan et al. 2015) and synthetic SAM analogues including TZD-C8 (Lidor et al. 2015) have been shown in vitro to reduce AHL production in various models. However, their specificity is limited because SAM participates in numerous essential cellular reactions, increasing the risk of off-target toxicity and reducing the clinical feasibility of non-selective inhibitors (LaSarre and Federle 2013). In addition, acyl chain analogs have been designed to interfere with acyl group incorporation by the enzyme (Shin et al. 2019), although the diversity of acyl-ACPs and the flexibility of the acyl-binding pocket complicate the development of universally effective inhibitors (Chan et al. 2015).

Another intervention route targets the availability of biosynthetic precursors (SAM and acyl-ACP). Compounds that interfere with acyl-ACP or fatty acid synthesis, such as triclosan, at sublethal concentrations inhibiting FabI (Hoang and Schweizer 1999), have been shown to reduce AHL production by limiting the acyl pool. Nonetheless, this mechanism lacks selectivity and may produce bactericidal effects or disturb essential metabolic pathways. Furthermore, many of these compounds are substrates of efflux pumps, which collectively limit their clinical utility (Chuanchuen et al. 2001).

Natural compounds (flavonoids, phenolics, terpenoids) have emerged as a been reported to interfere withrich source of inhibitors targeting LasI/RhlI. Flavonoids such as quercetin, baicalin, chrysin, and vitexin have been shown to decrease AHL production and AHL-regulated phenotypes (biofilm formation, pyocyanin, elastase) in vitro and cell-based assays (Das et al. 2016, 2017; Lima et al. 2023). Phenolic compounds such as methyl gallate also exhibit QS inhibitory activity, reducing virulence factor production and biofilm formation in *P. aeruginosa* (Naga et al. 2023). Additionally, plant-derived aldehydes such as trans-cinnamaldehyde have been reported to interfere with QS signaling and attenuate virulence-associated traits (Chang et al. 2014).These agents often act through weak-to-moderate enzyme interactions combined with pleiotropic effects on gene expression, making them attractive for their low cytotoxicity, though they usually require high concentrations and show strain-dependent variability.

From a translational perspective, several practical limitations hinder the progression of LasI/RhlI inhibitors toward clinical application: the redundancy and plasticity of the *P. aeruginosa* QS circuitry, low permeability and efflux-mediated extrusion of many synthetic inhibitors, metabolic side effects when targeting common precursors (SAM, acyl-ACP), and a lack of robust in vivo studies demonstrating efficacy without toxicity. Consequently, while conceptually promising, this strategy requires highly selective compounds and formulations capable of overcoming pharmacokinetic barriers (LaSarre and Federle 2013; Chan et al. 2015).

### Inhibition of PQS synthesis

The biosynthetic pathway of quinolone signals (HHQ and PQS) begins with anthranilic acid activation by the ligase PqsA, followed by condensation and modification reactions catalyzed by PqsD, PqsE, and the PqsBC complex, leading to HHQ formation. HHQ is subsequently oxidized by the flavin-dependent monooxygenase PqsH to generate PQS, the most potent regulator within this system. Within this cascade, PqsA, PqsD, and PqsBC represent major control points, as their inhibition significantly decreases HHQ and PQS levels, thereby disrupting QS signaling (Allegretta et al. 2017).

Due to the multistep organization of the PQS biosynthetic pathway and the diversity of enzymatic targets involved, the currently available inhibitors and their main characteristics are summarized in Table 2.

**Table 2:** Inhibitors targeting enzymes involved in PQS autoinducer biosynthesis. Only compounds with demonstrated or predicted specificity for each enzymatic target are included

Enzyme	Function	Representative inhibitors	Observed effect		Reference
PqsA	Anthraniloyl-CoA synthetase	Anthraniloyl-AMS Anthraniloyl-AMSN Halogenated anthranilic acid analogs Azorubine		Strong reduction of HHQ and PQS production in vitro (> 90% for AMS/AMSN); inhibition of PqsR-dependent virulence and reduced pathogenicity in vivo (mouse models); azorubine interferes with QS and reduces virulence-related traits in vitro	Lesic et al. 2007; Ji et al. 2016; Al-Shabib et al. 2020
PqsBC	Heterodimeric enzyme catalyzing conversion of 2-ABA to HHQ	2-AA Benzamide–benzimidazole derivatives		2-AA shows EC₅₀ = 46 µM (enzyme) and 319 µM (HHQ reduction) in vitro in heterologous systems; dual targeting potential (PqsR/PqsBC)	Drees et al. 2016; Maura et al. 2017
PqsD	Condensation enzyme forming the quinolone core (DHQ)	Anthraniloyl-CoA transition-state analogs 2-BB derivatives Ureidothiophenecarboxylic compounds		Up to 77% reduction in HHQ/PQS production and ~ 38% reduction in biofilm formation in vitro; structure-based design	Pistorius et al. 2011; Storz et al. 2012; Hinsberger et al. 2014; Sahner et al. 2015
PqsE	Regulatory hydrolase involved in virulence	-		Not directly targeted in PQS biosynthesis; central role in QS-regulated virulence	Discussed separately (Strategy V)
PqsH	Flavin-dependent monooxygenase converting HHQ to PQS	Natural compounds (e.g., CNP0000215, CNP0007440)		Predicted strong binding affinity to PqsH in silico; potential inhibition of PQS formation	Narthanareeswaran et al. 2025

### Inhibition of PqsA

PqsA, an anthraniloyl-CoA synthetase, catalyzes the initial step in quinolone signal biosynthesis by activating anthranilic acid to form anthraniloyl-AMP and then anthraniloyl-CoA (Allegretta et al. 2017). Although multiple classes of compounds targeting this enzyme have demonstrated the ability to reduce HHQ and PQS production, their translational potential varies significantly. Notably, halogenated anthranilic acid analogs have shown efficacy in vivo in murine infection models, resulting in reduced bacterial dissemination and increased host survival (Lesic et al. 2007), supporting the relevance of PqsA as a therapeutic target within the PqsR regulatory pathway. In contrast, anthraniloyl-AMP analogs such as anthraniloyl-AMS and anthraniloyl-AMSN exhibit strong enzymatic inhibition in vitro (Ji et al. 2016), but their applicability is limited by poor cellular permeability and lack of validation in complex biological systems.

Indirect modulators such as azorubine further illustrate the challenges associated with specificity, as their effects on QS appear to be context-dependent and mechanistically insufficiently defined (Al-Shabib et al. 2020). Compared to other nodes within the PQS pathway, targeting PqsA offers the theoretical advantage of blocking the initial step of signal biosynthesis. However, this upstream position may also require near-complete inhibition to achieve a measurable phenotypic effect, further complicating therapeutic application. Overall, while PqsA represents an attractive upstream target for disruption of quinolone signal biosynthesis, current inhibitory strategies are constrained by limited drug-like properties and incomplete understanding of intracellular target engagement, highlighting a significant gap between biochemical activity and in vivo therapeutic efficacy.

### Inhibition of PqsD

PqsD is a condensation enzyme responsible for forming the aromatic core of quinolones from anthraniloyl-CoA and malonyl-ACP, producing the intermediate 2,4-dihydroxyquinoline (DHQ) (Allegretta et al. 2017).

Initially, inhibitors were identified among compounds targeting FabH, an enzyme structurally and functionally related to PqsD, but these showed limited specificity and weak activity, highlighting the challenges of repurposing FabH-directed chemotypes for this system (Pistorius et al. 2011).

More recent efforts have yielded transition-state analogs of anthraniloyl-CoA, which demonstrated improved enzymatic inhibition and moderate reductions in HHQ and PQS production, as well as partial inhibition of biofilm formation (Storz et al. 2012). Despite these biochemical effects, the translation of these compounds into physiologically relevant conditions remains limited.

Structure-based approaches have identified two major inhibitor families, including 2-benzamidobenzoic acid derivatives (BB derivatives) (Hinsberger et al. 2014) and ureidothiophenecarboxylic scaffolds (Sahner et al. 2015) While these studies support the druggability of PqsD, their development is largely constrained to in vitro enzymatic assays and computational docking studies, with limited evidence of robust intracellular activity.

Importantly, inhibition of PqsD may also lead to the accumulation of upstream metabolic intermediates, which could potentially exert toxic effects or trigger metabolic rerouting, thereby complicating the interpretation of phenotypic outcomes.

Overall, although PqsD represents an attractive enzymatic node within the PQS biosynthetic pathway, current inhibitors are hindered by poor cellular penetration, likely active efflux, and insufficient validation under infection-relevant conditions.

### Inhibition of PqsE

Although PqsE is a hydrolase, its catalytic function is not essential for PQS biosynthesis. Instead, it plays a central regulatory role in virulence through its interaction with the Rhl system (Borgert et al. 2022). For this reason, it is discussed in detail under Strategy V.

### Inhibition of PqsB and PqsC

PqsB and PqsC form a heterodimeric enzyme complex with an unexpected architecture within the FabH-like family, as revealed by X-ray crystallography. Their main function is to catalyze the conversion of the precursor 2-aminobenzoylacetate (2-ABA) into HHQ, making this complex a critical control point in the biosynthetic route (Drees et al. 2016; Allegretta et al. 2017).

Although 2-aminoacetophenone (2-AA) has demonstrated inhibitory activity against PqsBC, its potency is modest, with micromolar EC₅₀ values observed in both purified enzyme assays and heterologous systems (Drees et al. 2016). The discrepancy between enzymatic and cellular activity suggests limited effective target engagement under more complex biological conditions, likely due to reduced intracellular accumulation or competition with native substrates. In addition, the relatively high concentrations required to achieve measurable inhibition raise concerns regarding pharmacological feasibility and specificity, particularly in the context of infection environments where substrate availability and metabolic flux may vary.

Interestingly, benzamide–benzimidazole derivatives initially designed as PqsR inhibitors have also been reported to interfere with PqsBC activity (Maura et al. 2017). This is a particularly attractive feature as it could enhance therapeutic efficacy and offer alternative targets to mitigate the emergence of resistance when focusing on a single molecular site. However, it also complicates mechanistic interpretation and raises questions about selectivity.

### Inhibition of PqsH

PqsH is a flavin-dependent monooxygenase that converts HHQ into PQS, the active form that interacts with the regulator PqsR. Because PqsH activity requires oxygen, microaerobic conditions naturally reduce PQS production (Schertzer et al. 2010).

In contrast to other enzymes in the PQS biosynthetic pathway, PqsH remains poorly characterized as a drug target. Although recent in silico and structural bioinformatics studies have identified potential ligands with predicted affinity for the enzyme, including compounds reported to interact with the FAD-binding region, these findings are currently limited to computational predictions and lack experimental validation in biochemical or cellular systems (Narthanareeswaran et al. 2025).

To date, no compounds have demonstrated robust efficacy in animal models or shown selective inhibition of the flavin-binding site of PqsH. Furthermore, since HHQ can also activate PqsR—albeit less efficiently than PQS—partial inhibition of PqsH may lead to HHQ accumulation and residual signaling, complicating the interpretation of therapeutic outcomes.

### Degradation or inactivation of autoinducer molecules

The degradation or inactivation strategy aims to prevent autoinducer molecules from reaching their receptors, thereby blocking the activation of QS-dependent virulence genes. Research has primarily focused on enzymes capable of modifying or destroying AHLs, although immunological approaches and synthetic sequestrants have also been explored.

Among the best-characterized QQ enzymes are lactonases, which hydrolyze the lactone ring of AHLs, thereby inactivating the signal and preventing receptor recognition. Since the foundational discovery of AiiA (Dong et al. 2000), which provided the first evidence that enzymatic signal degradation could attenuate bacterial virulence, the field has evolved from identifying diverse structural families—such as metallo-β-lactamase-like and phosphotriesterase-like lactonases—to optimizing their specificity and stability for therapeutic use (Sikdar and Elias 2020; Rémy et al. 2020).

Recent research emphasizes that lactonase specificity is a key determinant in the disruption of *P. aeruginosa* regulatory circuits, directly influencing virulence factor production and biofilm architecture (Rémy et al. 2020). For instance, engineered variants of the archaeal lactonase SsoPox (e.g., SsoPox-W263I) have demonstrated remarkable efficacy in quenching virulence in clinical isolates and can even modulate the expression of bacterial CRISPR-Cas systems (Mion et al. 2019). Furthermore, novel enzymes such as the YtnP family (Djokic et al. 2022; Curcic et al. 2024) and the marine-derived RmmLII (Shen et al. 2025) have shown high potency in reducing biofilm formation and enhancing antibiotic synergy in in vivo murine models, even against multidrug-resistant (MDR) strains.

The bridge between biochemical discovery and anti-virulence therapy is currently being strengthened by advanced delivery systems. Recent studies have successfully utilized lactonase-loaded hydrogels to control MDR *P. aeruginosa* infections in burn wound models (Sakr et al. 2021). Moreover, the identification of highly thermostable and robust enzymes (Porzio et al. 2023; Liu et al. 2026) reinforces the potential of lactonases as potent adjuncts to the current antibiotic arsenal, shifting the focus from endogenous eukaryotic defenses, such as mammalian paraoxonases (PONs), toward high-performance recombinant and environmental QQ agents (Sikdar and Elias 2020).

AHL-acylase enzymes are key QQ biocatalysts by irreversibly cleaving the amide bond of AHLs. This enzymatic degradation leads to broad alterations in gene expression, virulence, and biofilm development. Unlike lactonases, *P. aeruginosa* possesses endogenous acylases, such as PvdQ and QuiP, which are essential for signal turnover and metabolic adaptation (Wahjudi et al. 2011). Recent research has transitioned from characterizing these native enzymes to enhancing their clinical utility through advanced protein engineering. For instance, directed evolution has yielded PvdQ and MacQ variants with significantly improved kinetic and biochemical properties, overcoming the limitations of wild-type enzymes in therapeutic settings (Sompiyachoke and Elias 2024). These biocatalysts have been successfully incorporated into multifunctional coatings and anti-biofilm surfaces—such as catheters and PDMS silicone—to simultaneously inhibit bacterial adhesion and signal accumulation (Vogel et al. 2020; Zou et al. 2022).

Furthermore, the development of enzyme-based nanostructures and hybrid platforms, combining acylases with antimicrobial agents or photothermal therapy, has demonstrated enhanced efficacy in eradicating established MDR *P. aeruginosa* biofilms (Ivanova et al. 2022; Song et al. 2023). This diversity of QQ tools continues to expand with the identification of novel enzymes such as AhaP (Reina et al. 2021) and PoAcy1 (Liu et al. 2026), which exhibit potent anti-virulence activity and improve survival in pulmonary and systemic infection models.

Oxidoreductases represent a mechanistically distinct strategy by targeting PQS. Although the resulting molecules retain structural similarity to the native form, such alterations significantly reduce transcriptional activation of virulence genes. A prominent example is the mycobacterial dioxygenase AqdC, a cofactor-independent enzyme that inactivates PQS by cleaving its quinolone ring (Birmes et al. 2019). Recent protein engineering has significantly enhanced the stability and kinetics of AqdC, reinforcing its potential as a robust anti-virulence tool (Arranz San Martín et al. 2022; Wullich et al. 2021).

Furthermore, the link between redox biology and QS—involving enzymes like rubredoxin reductase (Wiehlmann et al. 2025) and nitrate reductases (Yan et al. 2019)—offers novel targets for metabolic interference and stress adaptation. Importantly, combined strategies utilizing AqdC in tandem with AHL-lactonases (e.g., QsdA) provide a synergistic attenuation of *P. aeruginosa* pathogenicity, offering a more comprehensive, multi-pronged approach than targeting single signaling circuits alone (Birmes et al. 2019).

Despite their potential, the clinical translation of QQ enzymes is hindered by two primary bottlenecks: proteolytic instability and host immunogenicity. The infection site, particularly in *P. aeruginosa* lung or wound infections, is a hostile environment rich in host and bacterial exoproteases (e.g., LasB elastase) that rapidly degrade exogenous proteins (Fetzner 2015). Moreover, the non-human origin of these biocatalysts poses a risk of triggering neutralizing antibodies, which can compromise both safety and long-term efficacy (Kaufmann et al. 2008a).

To address these limitations, current research has shifted from using free enzymes toward advanced delivery platforms. Encapsulation within biocompatible hydrogels or immobilization onto medical-grade surfaces effectively “masks” the enzymes from the host immune system and proteolytic attack while maintaining their catalytic activity (Sakr et al. 2021; Vogel et al. 2020). Moving forward, combining these protective scaffolds with engineered, hyper-thermostable variants represents the most viable path to transforming QQ into a robust adjunct to conventional antibiotic therapy.

Selected examples of QQ enzymes discussed here are presented in Table 3.

**Table 3:** Representative QQ enzymes acting through degradation or modification of autoinducers in *P. aeruginosa*

Type of Agent	Name	Model	Observed Results	Reference
Lactonase	SsoPox-W263I	In vitro (Clinical isolates)	20-fold increase in k_cat_/K_M_; 80% reduction in pyocyanin and protease activity.	Mion et al. 2019
	YtnP family	In vitro (Biofilm assays)	70–90% reduction in biofilm biomass and inhibition of swarming motility.	Djokic et al. 2022
	RmmLII	In vivo (Murine infection model)	65% reduction in biofilm; significant enhancement of antibiotic synergy in vivo.	Shen et al. 2025
	QsdA-Hydrogel	In vivo (Murine burn wound)	3-log reduction (99.9%) in bacterial load; accelerated re-epithelialization of the wound.	Sakr et al. 2021
Acylase	PvdQ/MacQ	In vitro (Kinetic assays)	50% improvement in catalytic efficiency for 3-oxo-C12-HSL vs. wild-type enzymes.	Sompiyachoke 2024
	Immobilized PvdQ	Ex vivo (PDMS Silicone/Catheters)	~ 60% decrease in biofilm coverage on medical-grade surfaces after 24 h.	Vogel et al. 2020
	PoAcy1	In vivo (*Galleria mellonella*)	75% reduction in elastase; significantly increased survival rates in the larvae model.	Liu et al. 2026
	Nanoscaffold	In vitro/In vivo (Abscess model)	99.9% eradication of established biofilms using chemo-photothermal synergy.	Song et al. 2023
Oxidoreductase	AqdC	In vitro (Biochemical assay)	Complete degradation of 100 µM PQS in < 30 min; 90% reduction in eDNA release.	Wullich et al. 2021
	AqdC + QsdA	In vitro (Co-culture)	Synergistic inhibition (> 95%) of elastase and rhamnolipids by targeting AHL and PQS.	Birmes et al. 2019
	Rubredoxin Red.	In vitro (Mutant phenotyping)	40% increase in sensitivity to H₂O₂; impaired survival under oxidative stress.	Wiehlmann 2025

Beyond enzymatic mechanisms, alternative strategies focus on the physical sequestration of signaling molecules to prevent receptor activation. Synthetic platforms, such as molecularly imprinted polymers (MIPs) (Ma et al. 2018)—specifically those synthesized from itaconic acid (IA) and 2-hydroxyethyl methacrylate (HEMA)—and various cyclodextrins (CDs) (Berkl et al. 2022), including methylated quaternary ammonium α- and β- CDs, have been engineered to capture target AHLs. These “molecular traps” effectively reduce the extracellular concentration of autoinducers, leading to a significant inhibition of biofilm. Another theoretical approach involves promoting the chemical or physical instability of signaling molecules to decrease their extracellular half-life; however, this strategy remains largely unexplored experimentally and lacks the targeted precision of other QQ methods (Uroz et al. 2009).

In parallel, immunological approaches offer a highly specific route for QQ through passive or active immunization. Monoclonal antibodies (mAbs) act as “molecular sponges,” sequestering signals before they trigger downstream virulence circuits. Key examples include the murine antibody RS2-1G9, which significantly reduces virulence factor production in vitro, and the chimeric sheep–mouse antibody HSL-2, which has demonstrated the ability to increase survival rates in in vivo murine models (Kaufmann et al. 2006, 2008b; Palliyil et al. 2014). Although still in the experimental stage, these immunological tools and sequestration compounds provide a robust, non-catalytic complement to traditional antimicrobial and enzymatic strategies, offering a highly targeted approach to disarming *P. aeruginosa*.

### Modification of autoinducer release

This strategy aims to limit the export of autoinducers or prevent them from reaching critical intracellular concentrations.

The release of autoinducers does not rely solely on passive membrane diffusion but is mediated by specialized mechanisms such as outer membrane vesicles (OMVs). These structures act as nanocarriers that encapsulate and release hydrophobic signaling molecules, particularly quinolones from the Pqs system, ensuring their stability against enzymatic degradation and promoting efficient distribution in the bacterial environment. Interfering with OMV biogenesis or function has thus emerged as a potential means to disrupt bacterial communication (Kulkarni and Jagannadham 2014; Florez et al. 2017).

A recently developed experimental approach involves the use of modified cyclodextrins (Table 4). These molecules integrate into the outer membrane and can alter its lipid organization, thereby affecting OMV biogenesis and release. In vitro studies have shown that this approach reduces vesicle release and consequently limits the dissemination of signaling molecules and virulence factors (Barnaby et al. 2019). However, perturbation of the bacterial membrane is a global, non-specific process, which may trigger unintended side effects, including altered permeability to antibiotics or activation of compensatory adaptive responses. Therefore, although promising, this strategy requires careful evaluation before any clinical translation.

**Table 4:** Compounds affecting autoinducer release

Group	Specific molecule	Main target	Observed effect	Reference
Modified cyclodextrin	Methyl-β-cyclodextrin	Outer membrane lipids	Destabilizes membranas, interferes with OMV formation	Barnaby et al. 2019
	Hydroxypropyl-β-cyclodextrin			
Peptidomimetic antibiotic	L27-11 (polymyxin B analogue)	LptD	Interferes with outer membrane assembly; inhibits OMV formation	Nityakalyani et al. 2010)
Conjugated phenone derivative	Z-ethylthioeninone	MexEF-OprN efflux pump	Induces MexEF-OprN expression; reduces intracellular levels of autoinducers	Kristensen et al. 2024

Another approach involves peptidomimetic antibiotics targeting essential proteins involved in outer membrane biogenesis, such as LptD (Table 4). Since this protein is indispensable for lipopolysaccharide translocation, its inhibition indirectly affects OMV formation and, consequently, the release of autoinducers. Although these compounds have shown activity against *P. aeruginosa*, their capacity to modulate QS signaling independently of bactericidal effects remains to be clearly established (Nityakalyani Srinivas et al. 2010).

Finally, it has been reported that efflux pumps, particularly MexEF-OprN, contribute to the secretion of signaling molecules such as AHLs and PQS precursors. Chemical activation of this system can reduce the intracellular concentration of autoinducers and impair QS communication (Table 4). However, overexpression of MexEF-OprN is also associated with multidrug-resistant phenotypes, limiting its therapeutic applicability and posing a dilemma between QS modulation and antibiotic resistance (Mazza et al. 2024; Kristensen et al. 2024).

Overall, the manipulation of autoinducer release represents an innovative approach to interfere with bacterial communication. Nonetheless, its evaluation must carefully consider the specificity of the intervention, potential off-target effects on cellular processes unrelated to QS, and the risk of secondary resistance. Additional in vivo studies and the development of highly selective modulators will be essential to determine the feasibility of these strategies as adjuncts to conventional antibiotic therapy.

### Signal receptor inhibitors

Receptor inhibition represents one of the most promising approaches for the development of antivirulence therapies against *P. aeruginosa*. Unlike strategies focused on autoinducer degradation or disruption of their biosynthesis, receptor blockade directly prevents the translation of the signal into physiological responses. In this context, the three main receptors (LasR, RhlR, and PqsR) have been consolidated as therapeutically relevant targets, with a wide diversity of inhibitors described from both natural and synthetic origins.

In general, the activity of inhibitors has been mainly evaluated through gene expression analyses, including RNA extraction and microarrays, RNA sequencing (RNA-seq), quantitative real-time PCR, reporter gene assays, and molecular docking approaches (Li et al. 2022).

### LasR receptor inhibition

LasR inhibition represents a cornerstone of anti-virulence therapy, as this receptor serves as the master regulator of the Quorum Sensing (QS) network in *P. aeruginosa*. Unlike traditional bactericidal approaches, blocking LasR attenuates the expression of pathogenicity factors without compromising cellular viability, thereby reducing the selective pressure that typically leads to conventional resistance.

### Natural LasR inhibitors

The search for bioactive molecular scaffolds has found an inexhaustible source of chemical diversity in natural products, particularly plant-derived secondary metabolites such as phenolic compounds, flavonoids, essential oils, and organosulfur derivatives. Amon g the latter, garlic extracts containing vinyl-dithiin derivatives have demonstrated a specific ability to inhibit LasI/LasR signalling (Cady et al. 2012). Similarly, natural isothiocyanates found in cruciferous vegetables, such as sulforaphane and erucin, act by effectively blocking the interaction between the receptor and its native autoinducer (Ganin et al. 2013).

Essential oils have also emerged as potent anti-QS agents. For instance, *Murraya koenigii* oil interferes with the production of LasR-regulated pigments and siderophores, while 1,8-cineole—the primary component of niaouli oil—blocks autoinducer binding to the receptor, significantly limiting biofilm formation (Bai and Vittal 2014). In this vein, ginger derivatives, specifically 6-gingerol, have shown remarkable activity through a mechanism of partial structural mimicry with acyl-homoserine lactones (AHLs), directly interfering with LasR-dependent transcription (Vijendra Kumar et al. 2014).

Within the flavonoid family, molecules such as quercetin and naringenin have consolidated their relevance by competing directly with the (OdDHL) signal for the receptor binding site, resulting in a drastic reduction in AHL synthesis and virulence gene expression. Other compounds, including catechin-7-xyloside, saponol, butein, and cardamonin, have confirmed this affinity through docking studies and cellular assays (Hernando-Amado et al. 2020; Zhong et al. 2020; Baburam et al. 2022). Additionally, products like furvina, isolated from sugarcane bagasse (Borges et al. 2017), and trans-anethole from anise reinforce the potential of natural products as a foundation for the rational design of anti-virulence drugs (Hançer Aydemir et al. 2018).

### Synthetic LasR inhibitors

The design of synthetic inhibitors has evolved to overcome limitations of chemical stability and solubility associated with natural compounds, initially focusing on the creation of structural analogs of AHLs. These analogs generally retain the homoserine lactone (HSL) core or utilize equivalent heterocyclic lactones, while introducing strategic modifications to the acyl chain length or terminal functional groups (Duplantier et al. 2021). Several groups are summarized in Table 5.

**Table 5:** Inhibitors acting on the LasR receptor

Source		Molecule	Reference
Natural		Organosulfur compounds: vinyl dithiins (garlic) and isothiocyanates (broccoli)	Cady et al. 2012; Ganin et al. 2013
		Essential oils: *Murraya koenigii* oil; 1,8-cineole	Bai and Vittal 2014
		Phenolic compounds from ginger: 6-gingerol, 6-shogaol, zingerone	Vijendra et al. 2014
		Flavonoids: quercetin and naringenin	Baburam et al. 2022; Hernando-Amado et al. 2020; Zhong et al. 2020
		Furvina	Borges et al. 2017
		Trans-anethole	Aydemir et al. 2018
Synthetic	**Analogs**	Cyclic amine derivatives	Ishida et al. 2007; Smith et al. 2003
		Aromatic amine derivatives	Hodgkinson et al. 2012
		HSL-core derivatives	Liu et al. 2019; Amara et al. 2016; Geske et al. 2005
		HCL-core derivatives	McInnis and Blackwell 2011
		3-amino-2-oxazolidinone derivatives acylated with variable chains	Jiang et al. 2020
	**Non-analogs**	Halogenated furanones	Chang et al. 2019; Hentzer et al. 2002; Wu et al. 2004
		Synthetic phenacyl derivatives	Müh et al. 2006a, b
		2-alkyltetrazoles	Müh et al. 2006a, b
		Synthetic triphenyl derivatives	Mü et al. 2006
		2-methoxy-4-vinylphenolate	Shah et al. 2019
		Triazole–benzimidazole hybrids	Srinivasarao et al. 2019

Research has demonstrated that acyl chain length is a determining factor in inhibitory potency, particularly in derivatives containing cyclic amines, where longer chains favor higher affinity for LasR and RhlR (Ishida et al. 2007). Subtle modifdevelopment of antivirulenceications, such as hydroxyl or carbonyl substitutions at specific positions, can drastically alter activity, even transforming an agonist into an antagonist. A representative example is the hydroxycyclopentyl-ring analog, which can simultaneously block both receptors (LasR/RhlR), significantly reducing the production of elastase, pyocyanin, and biofilm robustness (Smith et al. 2003) Additionally, aromatic analogues have stood out for their anti-pyocyanin activity, underscoring the importance of optimizing hydrogen-bond interactions within the receptor pocket (Hodgkinson et al. 2012).

Modified HSL-core derivatives have provided more specific inhibitors. Some molecules irreversibly bind to key receptor residues, resulting in permanent inactivation and improved infection control in biological models (Amara et al. 2016). Urea-linked derivatives have shown multi-target activity, simultaneously inhibiting virulence processes such as rhamnolipid production and biofilm formation at micromolar concentrations (Liu et al. 2015). On the other hand, thiolactone analogs (HCL core) have exhibited potent antagonistic effects in heterologous systems, although their efficacy in *P. aeruginosa* remains an area for optimization due to variable selectivity.

To explain these behaviors, two mechanistic models have been proposed: the “cooperative agonist” mode, where the ligand allows the formation of partially active dimers, and the “bimodal ligand” mode, where the formation of inactive heterodimers results in more robust transcriptional inhibition (McInnis and Blackwell 2011). A notable advancement in this area is the development of oxazolidinone derivatives, which not only inhibit classic virulence factors but also act synergistically with antibiotics like meropenem, increasing bacterial sensitivity within the biofilm and improving survival rates in infection models. Despite the challenges posed by LasR’s high specificity, the use of alternative scaffolds such as cyclic amines or aromatic groups provides greater stability against hydrolysis and broader biological activity (Jiang et al. 2020).

#### Non-AHL analogs

The search for molecules structurally distinct for molecules structurally distinct from AHLs has identified compound families that overcome the pharmacokinetic barriers of classic analogs. Synthetic furanones, inspired by marine metabolites, were pioneers in demonstrating protective effects in lung infection models by reducing LasR-regulated gene expression (Hentzer et al. 2002; Wu et al. 2004). More recent versions of these furanones have shown potent inhibition of biofilms and pigment production combined with low cytotoxicity (Chang et al. 2019).

Another relevant group consists of phenacyl and tetrazole derivatives identified through high-throughput screening, which exhibit values in the micromolar to nanomolar range, ranking among the most effective LasR inhibitors described to date (Müh et al. 2006a, b).Similarly, triphenyl derivatives have been highlighted for their superior chemical stability and ability to establish key interactions with specific residues in the LasR binding site, confirmed by molecular docking (Mü et al. 2006).

Finally, triazole–benzimidazole hybrids and phenolate salts offer attractive alternatives due to their high solubility under physiological conditions and dual inhibitory capacity on both LasR and RhlR (Srinivasarao et al. 2019). These compounds leverage aromatic stacking interactions within the receptor to achieve exceptional levels of inhibition (Shah et al. 2019). Collectively, non-AHL analogs not only expand structural diversity but also provide pharmacokinetic and stability properties that position them as ideal candidates for the development of future clinical therapies.

### RhlR inhibition

Regulation of virulence through this receptor is particularly complex due to the repressive function that RhlR exerts on the Pqs system. The development of antivirulence inhibitors targeting this receptor includes both negative modulators, aimed at shutting down Rhl signaling, and partial or positive agonists, whose secondary effect is the indirect inhibition of the Pqs system (Table 6) (Duplantier et al. 2021; Li et al. 2022).

**Table 6:** Inhibitors acting on the RhlR receptor

Source	Molecule	Reference
Natural	Polyphenols: ellagic acid derivatives, tannic acid, rosmarinic acid	Ouedraogo and Kiendraogo 2016; Naik and Mahajan 2013; Fernández et al. 2018
Synthetic	8-hydroxyquinoline derivatives	Qiu et al. 2019
	Gingerol derivatives	Nam et al. 2020
	LasR inhibitors with affinity for RhlR: N-cycloalkylamine and N-arylamine analogs	Ishida et al. 2007; Smith et al. 2003; Hodgkinson et al. 2012

Certain RhlR analogs with partial agonist profiles have shown a dual effect of particular interest: on one hand, they reduce pyocyanin synthesis by indirectly repressing the *pqs* system; on the other, they decrease the ability of *P. aeruginosa* to form mature biofilms. In vivo assays have demonstrated that this behavior translates into improved survival in infected models, supporting the functional relevance of this modulation (O’Loughlin et al. 2013; Eibergen et al. 2015). However, by activating RhlR, these compounds also stimulate rhamnolipid production—molecules that promote bacterial motility and biofilm dispersal—potentially enhancing the ability to colonize respiratory epithelium. This balance between beneficial effects (reduction of toxic pigments and early biofilm formation) and counterproductive effects (promotion of bacterial dissemination) illustrates the complexity of using RhlR partial agonists as an antivirulence strategy (Zulianello et al. 2006).

On the other hand, specific antagonists with 8-hydroxyquinoline-based structures have been described; these compounds significantly reduce rhamnolipid and biofilm production without affecting pyocyanin levels. This selectivity is particularly appealing as it allows the modulation of specific virulence pathways without disturbing others, opening the door to the design of more refined and safer inhibitors (Qiu et al. 2019).

Finally, synthetic gingerol-based hybrid compounds have been shown to inhibit both biofilm formation and rhamnolipid production, with certain analogs bearing modifications in the ketone moiety acting as particularly potent antagonists(Nam et al. 2020). In addition to these synthetic compounds, several natural modulators have been identified. Among them, phenolic derivatives such as ellagic acid(Ouedraogo and Kiendrebeogo 2016), tannic acid(Naik and Mahajan 2013) and rosmarinic acid (Fernández et al. 2018)stand out for their ability to reduce the expression of RhlR-regulated genes or directly interfere with ligand–receptor binding.

#### PqsR inhibition

Inhibition of the PqsR receptor (also known as MvfR) represents a highly strategic approach to anti-virulence therapy, primarily because the *Pqs* system is virtually exclusive to *P. aeruginosa*. This specificity allows for the targeted attenuation of pathogenicity without disrupting the commensal microbiota that rely on more common signaling systems, such as the AHL-based circuits. PqsR coordinates critical pathogenic phenotypes, and its blockade has been explored through a diverse array of natural and synthetic modulators. Among natural inhibitors, microbial metabolites like stigmatellin Y (Boopathi et al. 2017) and plant-derived flavonoids such as wogonin (Wang et al. 2021) compete directly with endogenous quinolones for the PqsR binding pocket. Other phytochemicals, including the sesquiterpene alcohol farnesol (Li et al. 2020) and perillaldehyde (Benny et al. 2022), act by repressing the transcription of the *pqsABCDE* and *pqsH* operons or by direct protein interaction, ultimately leading to a significant reduction in pyocyanin biosynthesis and biofilm formation.

The development of synthetic inhibitors has largely focused on designing analogs of the native autoinducers, PQS and HHQ. Structural modifications of the quinolone scaffold have yielded potent antagonists (Lu et al. 2012), such as carboxamide-bearing derivatives that effectively reduce pyocyanin synthesis (Kamal et al. 2017). Notably, some of these compounds act as “inverse agonists,” suppressing both ligand-induced activation and the receptor’s basal constitutive activity, which has been shown to protect hosts in animal infection models (Lu et al. 2014). Other successful scaffolds include quinazolinones, where the replacement of the characteristic PQS hydroxyl group with a primary amine converts agonist activity into antagonism (Grossman et al. 2020). Furthermore, 4-aminoquinoline derivatives substituted with chloro or trifluoromethyl groups have demonstrated a dual capacity to inhibit pyocyanin secretion (Aleksić et al. 2017) and biofilm formation (Ilangovan et al. 2013), often showing synergy with conventional antibiotics like tobramycin to disrupt mature biofilms (Soukarieh et al. 2018). More recent research into alternative heterocyclic cores, such as aminopyridines (Zender et al. 2020), pyranones (Li et al. 2018), and pyridinones (Liu et al. 2020), continues to confirm the structural versatility of the PqsR binding pocket. Although only a few candidates have demonstrated efficacy in vivo, the development of PQS and HHQ analogs has enabled clear structure–activity relationship mapping, identifying key substitutions that convert a natural agonist into an effective antagonist.

A significant shift in the field has been the exploration of non-analog scaffolds (Table 7), which offer superior pharmacological profiles and chemical distance from native quinolones. Hybrid benzamide derivatives with benzimidazole-based structures have emerged as particularly promising candidates. The lead compounds in this series exhibit submicromolar activity against PQS/HHQ production and remain effective against multidrug-resistant clinical isolates. Mechanistically, these inhibitors function through non-competitive binding, inducing a conformational change that inactivates the receptor and halts the downstream transcriptional cascade (Starkey et al. 2014). While related hybrid benzamide–triazinoindole compounds achieve comparable potency, they often lack the robust ability of benzimidazoles to reverse antibiotic tolerance in biofilms (Soukarieh et al. 2020). Additionally, indole-naphthalene hybrids have shown strong in vivo efficacy and retain their activity after oral administration, suggesting high potential for versatile clinical formulations (Hanot et al. 2024). Finally, small hydrophilic molecules, such as benzhydroxamic acid (Klein et al. 2012) and oxadiazole derivatives (Zender et al. 2013), serve as critical starting points for structural optimization, as they maintain activity despite the formidable permeability barrier of the *P. aeruginosa* outer membrane.

**Table 7:** Inhibitors acting on the PqsR receptor

Source		Molecule	Reference
Natural		Stigmatellin Y	Boopathi et al. 2017
		Wogonin	Wang al. 2021
		Farnesol	Li et al. 2020
		Perillaldehyde	Benny et al. 2022
Synthetic	**Analogs**	Quinolone scaffold	Lu et al. 2012; Kamal et al. 2017; Lu et al. 2014
		Quinazoline scaffold	Ilangovan et al. 2013; Grossman et al. 2020
		4-aminoquinoline scaffold	Aleksić et al. 2017; Soukarieh et al. 2018
		Pyridine, pyranone, or pyridinone scaffold	Zender et al. 2020; Li et al. 2018; Liu et al. 2020
	**Non- analogs**	Benzamide–benzimidazole hybrids (e.g., M64)	Starkey et al. 2014
		Benzamide–triazinoindole hybrids amida-triazinoindol	Soukarieh et al. 2020
		Aryl-oxazetoindole family	Hanot et al. 2024
		Benzhydroxamic acid and 2-amino-oxadiazole derivatives	Klein et al. 2012; Zender et al. 2013

#### Inhibition of the downstream signaling cascade

The final strategy to interfere with QS consists of blocking the effector phase that follows receptor activation, thereby preventing the perceived signal from being translated into specific physiological responses. This approach offers the advantage of markedly reducing pathogenic impact even after autoinducer detection has already occurred.

To date, only a few exogenous molecules have been unequivocally identified as inhibitors of this step in *P. aeruginosa* (Li et al. 2022; Rather et al. 2022) However, studies in other microorganisms have described compounds capable of preventing the interaction of the receptor–autoinducer complex with the promoter regions of target genes, thus blocking the transcription of key virulence genes. Other approaches rely on molecules that alter the stability of the transcriptional complex, inducing inactive conformations or promoting its proteolytic degradation, thereby preventing its action on DNA (Zhou et al. 2020; Duplantier et al. 2021).

In addition, indirect strategies are being explored, such as inhibition of transcriptional cofactors or accessory proteins, and even the introduction of heterologous regulators that compete for DNA-binding sites, reducing the expression of virulence-associated genes (Gökalsın et al. 2017; Rather et al. 2022; Qiu et al. 2024). In this regard, the PqsE protein enhances the affinity of RhlR for its target promoters, promoting virulence gene expression and modulating biofilm architecture (Borgert et al. 2022). High-throughput screening of FDA-approved drugs has yielded promising candidates, including nitrofurazone and erythromycin estolate, which prevent this interaction and were shown to reduce toxin and rhamnolipid production as well as bacterial motility. However, issues of toxicity and the lack of clarity regarding their precise mechanism of action currently limit their clinical potential (Baldelli et al. 2020).

## Current applications and future perspectives

The future of targeted therapies against *Pseudomonas aeruginosa* is grounded in a paradigm shift from traditional bactericidal approaches toward anti-virulence strategies, specifically through the inhibition of QS systems (Al-Maddboly et al. 2025). As a critical opportunistic pathogen, *P. aeruginosa* utilizes complex hierarchical networks, comprising the Las, Rhl, PQS, and IQS systems, to coordinate the expression of virulence factors, including toxin release and biofilm formation, which confer exceptional intrinsic and acquired resistance (Vadakkan et al. 2024a). Future perspectives suggest that blocking these cellular communication pathways allows for the attenuation of pathogenicity without directly affecting bacterial viability. This approach minimizes selective pressure and, consequently, limits the development of novel resistance mechanisms (Vadakkan et al. 2024b).

Current QQ strategies are primarily categorized into enzymatic degradation of autoinducers, the use of naturally derived inhibitors (phytochemical and marine), and the repurposing of approved drugs (Vadakkan et al. 2024a). Various QQ enzymes, such as acyl-homoserine lactonases and acylases, have been investigated for their ability to hydrolyze signaling molecules like AHLs. For instance, the acylase PvdQ has significantly reduced virulence in murine pulmonary infection models (Vadakkan et al. 2024b). Also a wide range of secondary metabolites with anti-QS activity has been documented, including quercetin, naringenin, baicalin, curcumin, and 6-gingerol (Vadakkan et al. 2024b). Recent studies highlight galloylquinic acids (GQAs) from *Copaifera lucens*, which potently inhibit *lasI/lasR* and *pqsA/pqsR* genes, thereby promoting the healing of infected wounds (Al-Maddboly et al. 2025).Isoliquiritigenin, a natural chalcone, acts as a multi-target inhibitor that enhances the pathogen’s sensitivity to aminoglycosides (Song et al. 2025). Marine-derived compounds, such as eicosyl heptafluorobutyrate (EPB) from *Shewanella indica*, represent a new class of agents with high affinity for the LasR receptor and promising pharmacokinetic potential (Shah et al. 2025). Furthermore, FDA-approved drugs like sitagliptin, metformin, and ceftriaxone (in complex with nickel) have been reevaluated for their capacity to displace natural ligands from QS receptors. In the synthetic realm, AHL analogs like YXL-13 have shown efficacy even against clinical isolates harboring mutations in the master regulator *lasR*. Complementing these chemical strategies, nanotechnology, for example using gold, silver, or chitosan nanoparticles, enables the controlled release of inhibitors such as ajoene or cinnamaldehyde, improving their stability and penetration into biofilms (Wang et al. 2026).

Despite this potential, anti-QS therapies face critical barriers to clinical implementation, notably specificity and biological stability. Many inhibitors may interfere with the host’s beneficial microbiota or lack a sufficiently broad spectrum of action across all *P. aeruginosa* strains. Biological stability remains a recurring issue, as compounds may be rapidly degraded by host enzymes or fluctuations in pH and temparature within the infection site (Jadhav 2026). Furthermore, the metabolic redundancy of *P. aeruginosa* presents a significant challenge; bacterial populations can develop compensatory mutations, such as those in *lasR*, which reorganize the QS hierarchy and allow systems like Rhl to maintain virulence (Vadakkan et al. 2024b). There is also concern that, while QQ exerts less pressure than conventional antibiotics, bacteria may still develop resistance through altered receptor affinity or increased efflux capacity (Jadhav 2026).

The primary advantage of these therapies lies in their ability to act synergistically with conventional antibiotics, weakening biofilm architecture and restoring bacterial susceptibility to drugs that were previously ineffective. By being non-bactericidal, they offer a more sustainable approach to the global antimicrobial resistance (AMR) crissis (Vadakkan et al. 2024b). The future of this discipline is oriented toward personalized medicine, utilizing advanced genomic and proteomic analyses to design tailored treatment plans based on a patient’s specific microbial profile. The development of genetically modified microorganisms to produce QQ agents in situ, alongside targeted nanocarriers, is expected to revolutionize the management of chronic infecctions, such as those observed in cystic fibrosis patients (Vadakkan et al. 2024b). Integrating these novel inhibitors into conventional clinical protocols promises to reduce hospitalization rates and significantly improve public health outcomes.

## Data Availability

No datasets were generated or analyzed during the current study.
